# Anodal Transcranial Direct-Current Stimulation Over the Right Dorsolateral Prefrontal Cortex Influences Emotional Face Perception

**DOI:** 10.1007/s12264-018-0242-6

**Published:** 2018-06-14

**Authors:** Li-Chuan Yang, Ping Ren, Yuan-Ye Ma

**Affiliations:** 1 Laboratory of Brain and Cognition, Kunming Institute of Zoology, Chinese Academy of Sciences, Kunming, 650223 China; 2 State Key Laboratory of Brain and Cognition, Institute of Biophysics, Chinese Academy of Sciences, Beijing, 100101 China; 3 University of the Chinese Academy of Sciences, Beijing, 100049 China; 4 School of Nursing, University of Rochester Medical Center, Rochester, NY 14642 USA

**Keywords:** tDCS, Facial expression, Emotion, Dorsolateral prefrontal cortex

## Abstract

The dorsolateral prefrontal cortex (DLPFC) is considered to play a crucial role in many high-level functions, such as cognitive control and emotional regulation. Many studies have reported that the DLPFC can be activated during the processing of emotional information in tasks requiring working memory. However, it is still not clear whether modulating the activity of the DLPFC influences emotional perception in a detection task. In the present study, using transcranial direct-current stimulation (tDCS), we investigated (1) whether modulating the right DLPFC influences emotional face processing in a detection task, and (2) whether the DLPFC plays equal roles in processing positive and negative emotional faces. The results showed that anodal tDCS over the right DLPFC specifically facilitated the perception of positive faces, but did not influence the processing of negative faces. In addition, anodal tDCS over the right primary visual cortex enhanced performance in the detection task regardless of emotional valence. Our findings suggest, for the first time, that modulating the right DLPFC influences emotional face perception, especially faces showing positive emotion.

## Introduction

The dorsolateral prefrontal cortex (DLPFC) is considered to play a crucial role in many high-level functions, such as working memory, decision-making, and self-control [1–4]. In addition, increasing numbers of studies have demonstrated that the DLPFC is also involved in normal and abnormal emotion-related brain networks, along with the amygdala and other subcortical structures [5, 6]. Pleasant words induce more activity in the bilateral DLPFC than unpleasant words [7]. And the DLPFC is activated by pleasant visual stimuli and reduced by unpleasant ones in a working memory task [8]. In an electroencephalographic (EEG) study, Godinho *et al.* found that the right DLPFC is involved in the emotional modulation of pain [9]. In addition, some studies have also found that the DLPFC is involved in cognitive tasks related to emotional faces [10, 11].

Even though many studies have shown that the DLPFC is important for emotional modulation and emotional memory, the contribution of each hemisphere is controversial. One theory is that the right hemisphere is predominantly involved in processing emotion, and the left hemisphere is specialized for cognitive processes [12, 13]. In a working memory-related functional magnetic resonance imaging (fMRI) study, although the left DLPFC showed a pattern of activity similar to the right DLPFC, the general pattern is consistent with a right-lateralization of face and emotional distracters [14]. A previous study has reported that patients undergoing antidepressant treatment for major depression showed significantly increased right DLPFC activity in response to unattended fear-related stimuli [15]. Another study found that patients with depression exhibit diminished right DLPFC activation and reduced right DLPFC-amygdala coupling during emotion regulation [16]. Most of the previous studies have shown that the DLPFC, especially the right DLPFC, is involved in emotion-related working memory tasks, but it is still not clear whether the DLPFC directly modulates the detection of emotional facial expressions.

Transcranial direct-current stimulation (tDCS) is a non-invasive technique that is widely used to treat many diseases and to study cognitive processes. Anodal tDCS is considered to facilitate brain activity, whereas cathodal stimulation suppresses it. Several studies have demonstrated that anodal tDCS over the DLPFC significantly improves the emotional state of patients suffering emotional disorders, such as depression and autism [17–19]. In addition, several studies have shown that anodal stimulation over the DLPFC improves probabilistic classification learning [20], declarative memory [21], working memory [22], driving behavior [23], and decision-making [24, 25], while diminishing risk-taking behavior [26]. Recently, Penolazzi *et al.* reported a significant effect of tDCS on emotional memory: right anode/left cathode selectively facilitates the memory of pleasant images whereas left anode/right cathode selectively facilitates the memory of unpleasant ones [27]. So far as we know, there is no evidence that the right DLPFC directly modulates the detection of emotional facial expression.

In the present study, we set out to investigate the role of the right DLPFC in emotional face processing using tDCS. Our aims were to investigate whether emotional face detection can be modulated by stimulating the right DLPFC and to test the right-hemisphere hypothesis. Since some previous studies have reported an after-effect of tDCS in emotion regulation [28, 29], we also examined this after-effect.

## Materials and Methods

### Participants

All participants were right-handed with normal or corrected-to-normal vision. Seventy-eight undergraduates were paid to participate in four experiments. The participants in each experiment were as follows: Experiment 1 (12 females and 11 males; mean age, 23 years, range 19–29), Experiment 2 (13 females and 4 males; 21 years, 19–24), Experiment 3 (10 females and 10 males; 25 years, 21–29), and Experiment 4 (9 females and 9 males; 22 years, 19–24). All participants were blind to the purpose of the experiments. Each participant was given instructions for the experiment and provided written informed consent in accordance with procedures approved by the Ethics Committee of the Kunming Institute of Zoology, Chinese Academy of Sciences. All experiments were conducted in accordance with the ethical standards of the 1964 Declaration of Helsinki.

### Apparatus and Stimuli

All experiments were conducted in a dimly-lit sound-attenuated room. Participants were seated 70 cm in front of a display and kept their head on the chin-rest during the entire experiment. Visual stimuli were presented on a 19-inch CRT display. The screen resolution was 1024 × 768, and the refresh rate was 100 Hz. Participants responded using a keyboard. The experimental programs were written with MATLAB 6.5 and Psychtoolbox 2.54, which were supplied by Institute of Biophysics, Beijing, China.

**Fig. 1:**
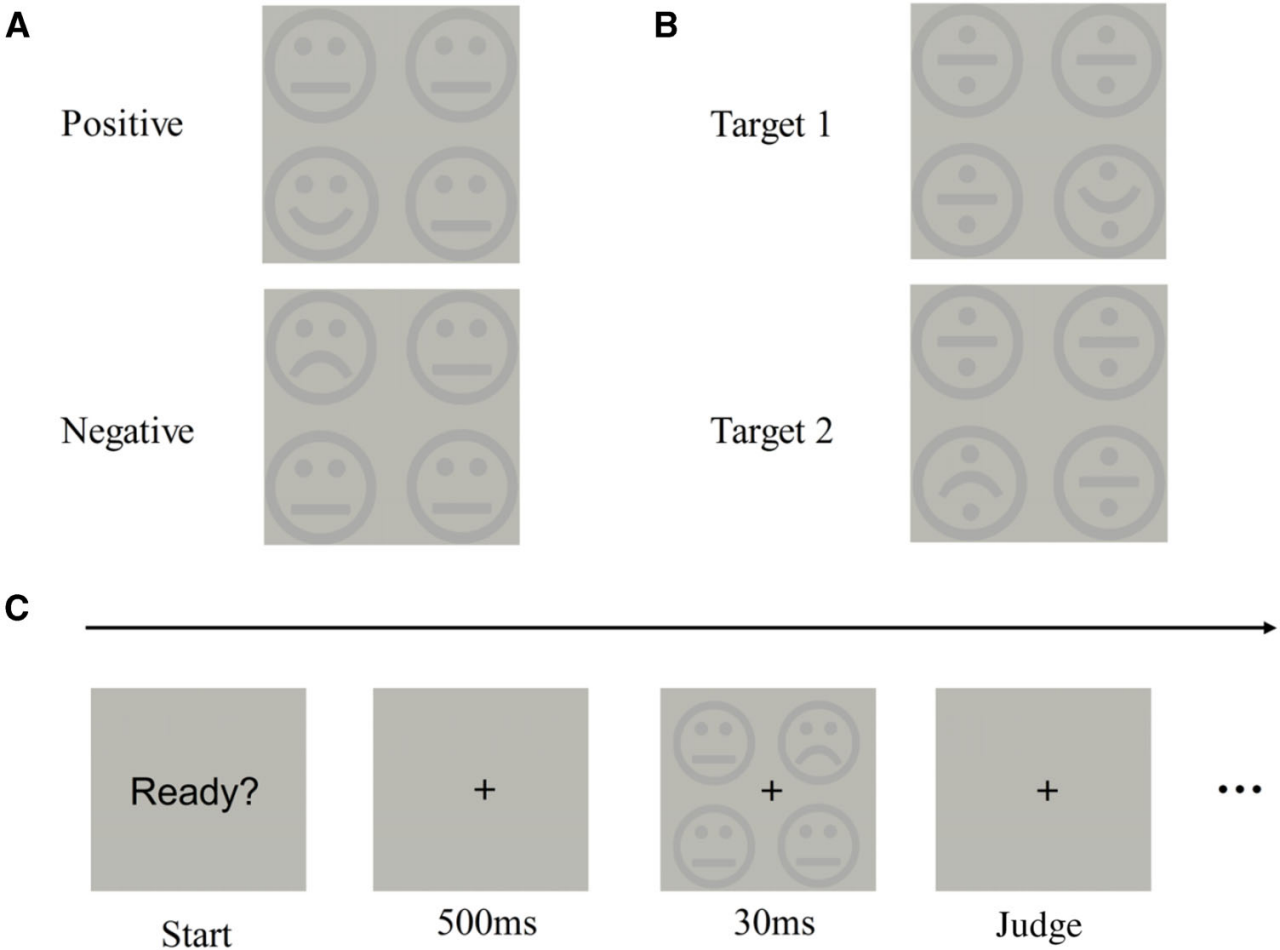
Emotional face detection task. **A** In Experiments 1, 2 and 3, the visual stimulus contained four schematic faces (one emotional and three neutral). **B** In Experiment 4, the visual stimulus was replaced by four scrambled faces. **C** The sequence of stimuli in the experimental procedure.

The visual stimuli contained four schematic faces (Fig. 1A) with two different conditions: one sad and three neutral faces, and one happy and three neutral faces. The visual angle of the image was 5.4°, and each schematic face was 2.5° with a 0.4° gap between them. In Experiment 4, the visual stimuli were replaced by scrambled faces (Fig. 1B). There were 6 different contrast levels of visual stimuli (relative to background): 1.1%, 2.2%, 3.3%, 4.4%, 5.6%, and 6.7%.

### Transcranial Direct-Current Stimulation

tDCS was delivered by a home-made battery-driven constant-current stimulator, at the State Key Lab of Brain and Cognitive Sciences, Institute of Biophysics, Beijing, China. The current was 1.5 mA and induced through two saline-soaked sponge electrodes (30 cm^2^ each). Previous studies have shown that a current intensity of no more than 2 mA is safe in normal participants. To locate the right DLPFC, the EEG 10/20 system is sufficient when using traditional tDCS electrodes, and has been widely used in many studies [30, 31]. In Experiments 1, 2, and 4, to stimulate the right DLPFC, the anode was placed over F4 (according to the EEG 10/20 system) and the cathode was over the supraorbital region. In Experiment 3, the anode was applied over O2 to stimulate the right primary visual cortex.

### Procedure

The stimulus sequence is illustrated in Fig. 1C. Each trial began with a “Ready?” placed in the center of the screen, and participants pressed the space-bar to start. After 500 ms fixation, the emotional faces were displayed for 30 ms, and then replaced by a blank screen. The participants were asked to press ‘5’ on the keypad if a sad face was displayed, and ‘6’ if a happy face was displayed. Before the formal test, there was a short practice with 10–20 trials to help participants become familiar with the task. In Experiment 1, there were 2 formal test sessions, Session 1 without tDCS and Session 2 with tDCS, which were designed to examine the direct effect of tDCS. Each session consisted of 240 trials, including 20 for each emotional face under each contrast level. There was a short rest (~10 min) between the two sessions. At the beginning of Session 2, the direct current was manually ramped up to 1.5 mA in 30 s. After 3 min adaptation to the stimulation, participants completed the same task as in Session 1. The experimental design was 2 (before and during tDCS) × 2 (sad and happy faces) × 6 (six contrast levels). Experiment 2 was designed to examine the after-effects of tDCS, and was essentially the same as Experiment 1 except for the following: after Session 1 in Experiment 2, the participants received tDCS stimulation for 15 min without a task; then Session 2 started immediately after cutting off the current, keeping the electrodes on the scalp until the rest of the experiment was finished. There were 336 trials in each part, with 28 trials in each condition. For comparison with the right DLPFC, the procedure of Experiment 3 was exactly the same as Experiment 1, except that the anodal electrode was moved to the right primary visual cortex (O2). In Experiment 4, all the schematic faces were scrambled to disrupt emotional information, and compared with the other three emotion-related experiments (Fig. 1B). The electrode locations and experimental design were exactly the same as in Experiment 1.

## Results

### Experiment 1

**Fig. 2:**
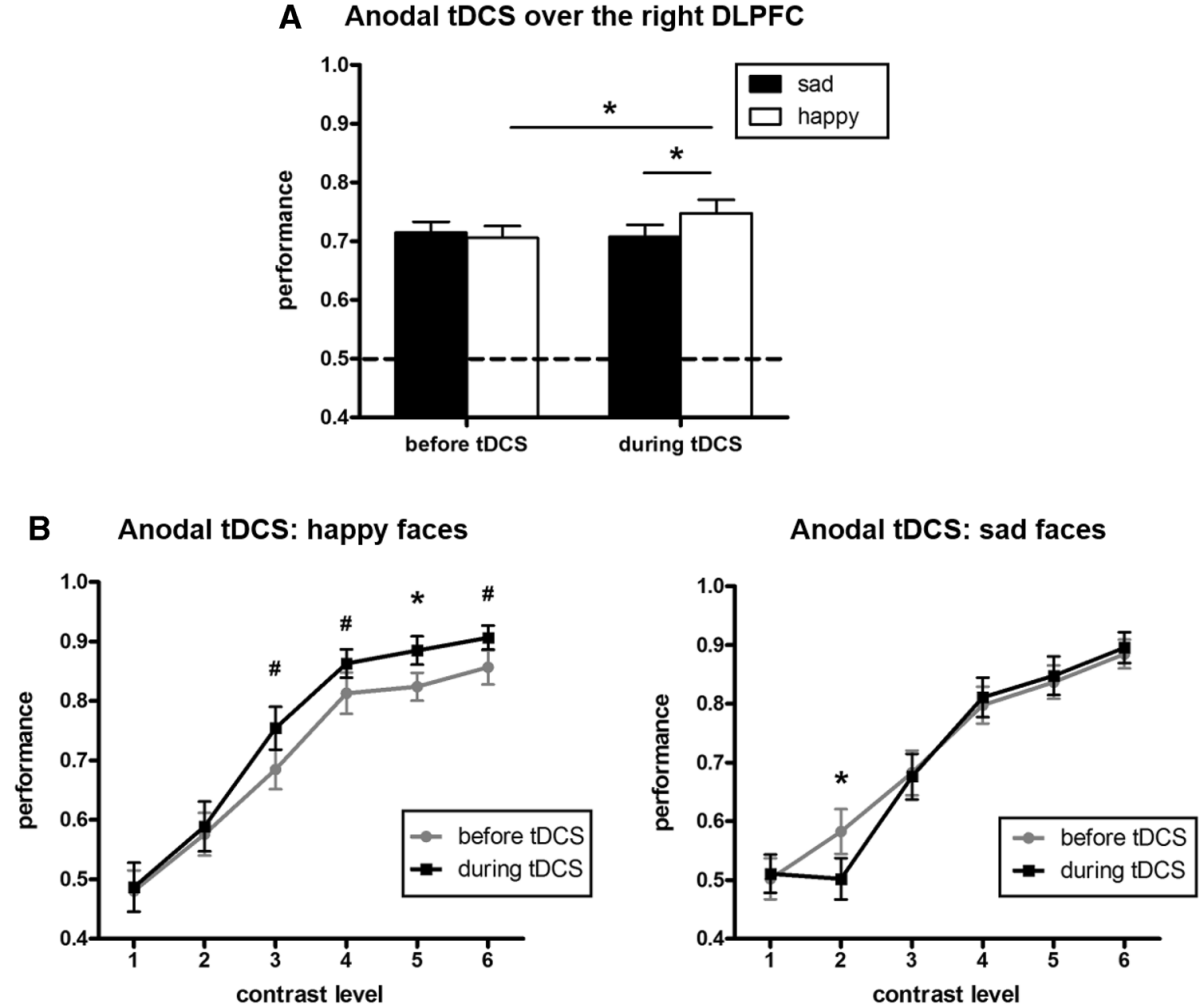
Performance in Experiment 1. **A** Performance was significantly better for a positive but not for a negative face, with the anode over the right DLPFC (dashed line, chance level of performance). **B** Average accuracy rates at each contrast level for positive and negative faces. (*P < 0.05; ^**#**^*P* < 0.1).

Incorrect responses were excluded before analysis. Repeated measures analyses of variances (ANOVAs) were used to analyze the accuracy data. A Greenhouse-Geisser correction was used to correct for unequal variances. The alpha level was 0.05 for all data analyses. There was a main effect of contrast level whereby better performance occurred with higher contrast levels [*F*(5, 110) = 117.636, *P* < 0.001]. Also, the interaction between tDCS and facial expression was significant [*F*(1, 22) = 7.448, *P* = 0.012; Fig. 2A]. After ANOVAs, paired *t*-tests were used to determine the significance details in each condition. *Post-hoc t*-tests showed that the accuracy of detecting a happy face was significantly higher during (74.75%) than before (70.58%) anodal tDCS (*P* = 0.036). However, the response to a sad face was not influenced by tDCS (before: 70.58%, during: 70.73%) (*P* = 0.569). During tDCS, the perception rate of the positive face was higher (74.75%) than that of the negative face (70.73%) (*P* = 0.029). Moreover, the performance was significantly better for a positive face during (88.48%) than before (82.39%) tDCS at contrast level 5 (*P* = 0.011). Similarly, the responses were slightly but not significantly better for a positive face during (75.43%, 86.30%, and 90.65%) than before (68.48%, 81.30%, and 85.65%) tDCS at contrast levels 3 (*P* = 0.071), 4 (*P* = 0.061), and 6 (*P* = 0.099) (Fig. 2B). For a negative face, the responses were significantly worse during (50.22%) than before (58.26%) tDCS only at contrast level 2 (*P* = 0.009).

### Experiment 2

**Fig. 3:**
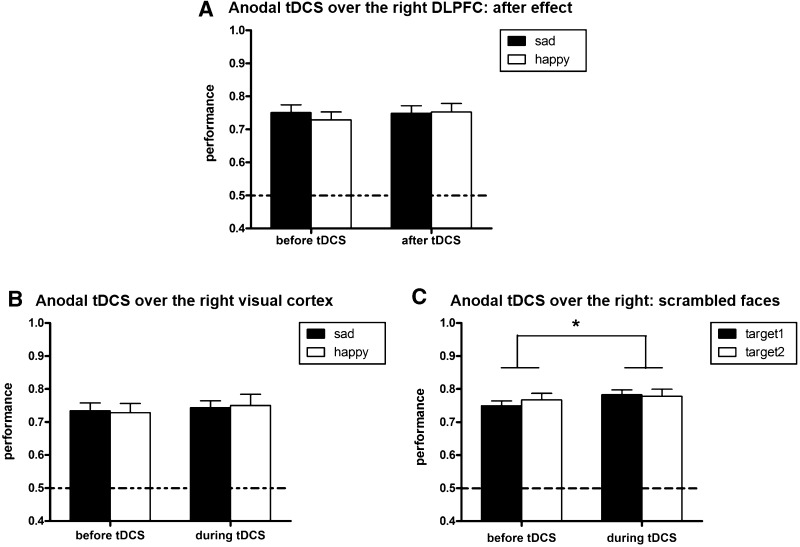
Performance in Experiments 2–4. **A** With the anode over the right DLPFC, there was no significant influence on emotional face perception after removing tDCS. **B** With the anode over the right primary visual cortex, there was only a slight tDCS effect on emotional face perception, with no difference between positive and negative faces. **C** Performance in the non-face task was significantly better when the anode was over the right DLPFC. (*P < 0.05).

The data analysis was the same as in Experiment 1. There was a main effect of contrast level, with better performance at higher levels [*F*(5, 80) = 114.037, *P* < 0.001]. The interaction between tDCS and facial expression was not significant [*F*(1, 16) = 1.621, *P* = 0.221] (Fig. 3A).

### Experiment 3

There was a main effect of contrast level, with better performance at higher levels [*F*(5, 95) = 119.848, *P* < 0.001]. The main effect of tDCS showed that anodal tDCS over O2 slightly enhanced the accuracy (before: 73.15%, during: 74.69%) [*F*(1, 19) = 4.263, *P* = 0.053]. The interaction between tDCS and facial expression was not significant [*F*(1, 19) < 1; Fig. 3B].

### Experiment 4

There was a main effect of contrast level with better performance at higher levels [*F*(5, 85) = 126.804, *P* < 0.001]. There was a main effect of tDCS, with better responses during (78.06%) than before tDCS (75.83%) [*F*(1, 17) = 4.761, *P* = 0.043]. The interaction between tDCS and facial expression was not significant [F (1, 17) = 2.566, *P* = 0.128; Fig. 3C].

## Discussion

In the current study, our results showed that tDCS is an effective and reliable tool to modulate brain activity. We found that anodal tDCS over the right DLPFC selectively facilitated the perception of positive but not negative faces. The effect of direct current only occurred during stimulation, and disappeared after removal of tDCS. The anodal stimulation over the right visual cortex also induced a slight enhancement of task performance with no difference between positive and negative faces. To the best of our knowledge, this is the first evidence of a relationship between the right DLPFC and emotional face detection using the tDCS technique.

In Experiment 1, using a two-forced-choice task, we investigated whether stimulating the right DLPFC could influence emotional face perception. Some previous tDCS studies have shown that the DLPFC plays an important role in the cognitive processes modulated by emotion [27] and several mental diseases (such as depression and pain) [18, 28, 29, 32]. In an fMRI study, Perlstein and colleagues showed that the right DLPFC is influenced by emotional pictures only in a working memory task but not in a detection task [8]. However, we found that the right DLPFC directly modulated performance in the emotional face detection task, which indicates that the DLPFC plays a more general role in processing emotional information regardless of the task. Different from the hypothesis of right hemisphere-emotion and left hemisphere-cognition, there is a valence-lateralization hypothesis which assumes that the right hemisphere is dominant for negative emotions and the left hemisphere is dominant for positive emotions [12, 33, 34]. Our results partly support the valence-lateralization hypothesis, except that the right DLPFC detects positive emotion. In line with our findings, Penolazzi *et al.* found that right anodal/left cathodal tDCS facilitates the recall of pleasant stimuli, whereas reversing the electrode polarity facilitates the recall of unpleasant stimuli [27]. fMRI studies have also reported that positive/pleasant stimuli promote more activity in the right DLPFC than negative/unpleasant stimuli [7, 8]. Since tDCS over the left DLPFC was not examined in the current study, it is difficult to speculate on hemispheric differences from our experimental design. In future studies, tDCS can be applied over the left and right DLPFC to compare the different effects in emotion detection. Compared with the anodal tDCS effect, cathodal tDCS is usually used to suppress brain activity. Although several studies have reported that the cathodal effects are not consistent and effective [22, 35, 36], it is worth examining the difference between cathodal and anodal effects on the DLPFC.

In Experiment 2, we tested the after-effect of tDCS using exactly the same stimuli as in Experiment 1. Some studies have reported that the effect of current over the DLPFC on working memory tasks can last several minutes to several hours after removing the tDCS [37, 38]. Improvement of emotion-related disorders has been found after anodal tDCS over the DLPFC [18]. However, we did not find a significant tDCS after-effect. Differing from those studies, we examined emotional face perception in the DLPFC after anodal tDCS, so the tDCS after-effect may be task-dependent. In addition, some may argue whether the tDCS effect is induced by the anodal electrode over the specific position (right DLPFC), or the cathode over the left supraorbital area. To clarify this problem, we moved the anode over the right primary visual cortex (O2) and kept the cathode over the left supraorbital region in Experiment 3. Compared with Experiment 1, the results showed a slight enhancement for both positive and negative faces. This demonstrated that a specific facilitation of positive faces was caused by stimulating the right DLPFC. And our result is congruent with Jolij’s finding that an emotional face task is disrupted by applying transcranial magnetic stimulation (TMS) to the visual cortex [39]. It is noteworthy that both emotion and localization tasks were disrupted by applying TMS to the visual cortex in Jolij’s study, indicating that the visual cortex is not specific to emotion tasks. To be consistent with Experiment 1, the stimuli in Experiment 3 were still displayed at the center of the screen, which is not the optimal location for activating the right primary visual cortex. Therefore, we would expect a stronger tDCS effect if the stimuli were displayed in the left visual field. Both Experiments 2 and 3 indicated that the selective facilitation of positive face perception is due to direct anodal tDCS over the right DLPFC.

Since we used schematic rather than natural faces, it may be argued that tDCS facilitated not emotional information processing but local features, such as mouth curvature. To exclude this possibility, we disrupted the configural information of faces by scrambling them in Experiment 4. In this way, the differences between target and distractors were only due to local features (such as mouth curvature). Our results showed no interaction of tDCS and targets, which means that the different effects of tDCS on positive and negative faces are based on emotional information but not local features. Interestingly, we found marginal significance of the tDCS main effect, which may be due to the possibility that the DLPFC plays an important role in visual attention as well, which has been reported in some human and animal studies [40, 41].

Finally, we need to acknowledge several limitations in the current study. First, we applied the cathode over the left supraorbital cortex, which is close to the orbitofrontal cortex. Comparing the right DLPFC and the visual cortex, the results demonstrated that the tDCS effect on detecting emotional faces was dependent on the anode over the right DLPFC. Nevertheless, the location of the cathodal electrode may influence tDCS as well. Using several electrode combinations, Antal *et al*. found that only the occipital-vertex (Oz-Cz) electrode position elicited a tDCS effect, implying that the direction of current flow should be considered [42]. Some previous studies have also shown that the orbitofrontal cortex is involved in emotion-related processes [8, 43]. In further studies, the cathode could be placed over other brain areas (such as Cz) to determine whether a change of current direction influences the effects. Second, although Experiments 2 and 4 can be used to confirm the direct tDCS effect on the right DLPFC, there was no traditional sham stimulation in the present study. To better control for the placebo effect, sham experiments should be considered in future research. Besides, there were more female participants in Experiment 2. Although several tDCS studies have reported that males and females do not differ in responding to stimulation in the frontal lobe [44, 45], a gender effect could be a potential factor in assessing the effects of tDCS in emotion-related tasks.

tDCS is an effective and reliable non-invasive technique for modulating brain function. We found that direct anodal tDCS over the right DLPFC selectively facilitated positive face processing compared with negative face processing. The results showed, for the first time, that modulating the DLPFC influences emotional face detection. In addition, the results support the hypothesis that the right hemisphere plays different roles in processing emotional information with different valences.
